# A Bundle of Services Increased Ascertainment of Tuberculosis among HIV-Infected Individuals Enrolled in a HIV Cohort in Rural Sub-Saharan Africa

**DOI:** 10.1371/journal.pone.0123275

**Published:** 2015-04-21

**Authors:** Frederick Haraka, Tracy R. Glass, George Sikalengo, Anna Gamell, Alex Ntamatungiro, Christoph Hatz, Marcel Tanner, Hansjakob Furrer, Manuel Battegay, Emilio Letang

**Affiliations:** 1 Bagamoyo Research and Training Centre, Ifakara Health Institute, Bagamoyo, United Republic of Tanzania; 2 Swiss Tropical and Public Health Institute, Basel, Switzerland; 3 University of Basel, Basel, Switzerland; 4 Ifakara Health Institute, Ifakara, United Republic of Tanzania; 5 Department of Infectious Diseases, Bern University Hospital Bern and University of Bern, Bern, Switzerland; 6 Division of infectious Diseases and Hospital Epidemiology, Departments of Medicine and Clinical Research, University Hospital Basel, Basel, Switzerland; 7 ISGlobal, Barcelona Ctr. Int. Health Res. (CRESIB), Hospital Clínic—Universitat de Barcelona, Barcelona, Spain; University of Cape Town, SOUTH AFRICA

## Abstract

**Objectives:**

To report on trends of tuberculosis ascertainment among HIV patients in a rural HIV cohort in Tanzania, and assessing the impact of a bundle of services implemented in December 2012, consisting of three components:(i)integration of HIV and tuberculosis services; (ii)GeneXpert for tuberculosis diagnosis; and (iii)electronic data collection.

**Design:**

Retrospective cohort study of patients enrolled in the Kilombero Ulanga Antiretroviral Cohort (KIULARCO), Tanzania.)

**Methods:**

HIV patients without prior history of tuberculosis enrolled in the KIULARCO cohort between 2005 and 2013 were included.Cox proportional hazard models were used to estimate rates and predictors of tuberculosis ascertainment

**Results:**

Of 7114 HIV positive patients enrolled, 5123(72%) had no history of tuberculosis. Of these, 66% were female, median age was 38 years, median baseline CD4+ cell count was 243 cells/µl, and 43% had WHO clinical stage 3 or 4. During follow-up, 421 incident tuberculosis cases were notified with an estimated incidence of 3.6 per 100 person-years(p-y)[95% confidence interval(CI)3.26-3.97]. The incidence rate varied over time and increased significantly from 2.96 to 43.98 cases per 100 p-y after the introduction of the bundle of services in December 2012. Four independent predictors of tuberculosis ascertainment were identified:poor clinical condition at baseline (Hazard Ratio (HR) 3.89, 95% CI 2.87-5.28), WHO clinical stage 3 or 4 (HR 2.48, 95% CI 1.88-3.26), being antiretroviralnaïve (HR 2.97, 95% CI 2.25-3.94), and registration in 2013(HR 6.07, 95% CI 4.39-8.38).

**Conclusion:**

The integration of tuberculosis and HIV services together with comprehensive electronic data collection and use of GeneXpert increased dramatically the ascertainment of tuberculosis in this rural African HIV cohort.

## Introduction

Tuberculosis (TB) remains the most common opportunistic infection among HIV-infected patients, in particular in sub-Saharan Africa (SSA), and can occur at any stage of HIV disease [1]. According to recent studies and reports from Tanzania, about 10% of all HIV-infected individuals develop active TB[2] whereas nearly half of TB patients are co-infected with HIV[3].

Trends of TB notification in Africa have decreased over time[4–6], which has been mostly attributed to improved screening and provision of antiretroviral therapy (ART) among TB-HIV infected individuals [5,6]. However, TB cases per population in SSA are still highest compared to other regions [4]. Lack of widely available point of care robust tools for TB diagnosis, high prevalence of HIV infection, and challenges in reporting of TB remain obstacles for TB control in SSA[4]. Moreover, diagnosis of TB in SSA often relies on the use of microscopy, radiological findings and clinical symptoms, and in most cases culture is not routinely performed due to minimal capacity and lack of infrastructure[7,8].

In December 2012, the Chronic Disease Clinic of Ifakara (CDCI) situated within the Saint Francis Referral Hospital (SFRH) in Ifakara, south-central Tanzania, introduced a bundle of services to optimize the functioning of the clinic in general and the diagnosis and management of TB-HIV cases in particular. This bundle of services consisted of three main components, namely the integration of TB and HIV services within one single facility, the use of GeneXpert (Cepheid, Sunnyvale CA) for TB diagnosis, and the introduction of an electronic data collection system.

Several studies have shown independent effectiveness of each of these strategies [9–22]. The WHO recommends integrating service delivery, use of point of care diagnosis, and replacing of paper clinic logs with electronic medical records. We hypothesized that the implementation of this bundle of services in our rural HIV cohort in Tanzania in December 2012 may have increased the rate of TB ascertainment.

## Methods

### Study design and setting

We conducted a retrospective cohort study using the KIULARCO cohort database between 2005 and 2013. This cohort is comprised of all patients visited at the Chronic Diseases Clinic of Ifakara, Morogoro, southern Tanzania, who give their informed consent to participate. Details of the KIULARCO cohort are given elsewhere[23,24].

### Participants

We included all patients enrolled in KIULARCO between 2005 and 2013. Exclusion criteria included:(i) reported history of TB; (ii) lack of follow-up information; and (iii) referral from a TB clinic. The KIULARCO studywasreviewed and approved by the IfakaraHealthInstitute (IHI) institutional reviewboard and the National Institute for MedicalResearch (NIMR) of Tanzania. Informed consent to participate in the KIULARCO cohort is sought from all patients upon registration in the Chronic Diseases Clinic of Ifakara. Regular supply of ART was introduced in our clinic in 2005.

### Variables and data source

#### Outcome

The outcome variable was TB recording in the KIULARCO database during the follow-up period, defined as having one of the following recorded in the database: 1) TB diagnosis, 2) positive sputum smear, 3) positive GeneXpert in sputum or other extra pulmonary sample 4) chest X-ray suggestive of TB together with at least one reported symptom of TB, or 5) having been treated with anti-tuberculosis drugs. We included both pulmonary and extra-pulmonary TB cases. Before the introduction of the electronic medical record in December 2012, data was collected using paper-based questionnaires filled by the clinicians at each visit and double data entered in anelectronic database by 2 data clerks. The structure of the questionnaire was entirely based on the patient record form of the Tanzanian National AIDS Control Program. Questions specifically concerning TB included screening questions (cough, weight loss, chronic fever, fatigue, night sweats), and TB diagnosis (yes/no/diagnosed today).

#### Predictors of tuberculosis ascertainment

The potential explanatory variables for tuberculosis ascertainment assessed included sex, age, marital status, baseline CD4 counts, WHO clinical stage, ART treatment, smoking, history of chronic illness (cancer, diabetes, hypertension), period of registration, and functional status. All variables were obtained from the KIULARCO cohort database.

### Definitions

#### Integration of TB and HIV within one facility

Before the introduction of the bundle of services, the TB and HIV care and treatment services at Saint Francis Referral Hospital were running in parallel and patients were referred between the two facilities. Integration of TB and HIV services included bringing all services under one facility. After the start of the bundle of service HIV patients are systematically screened for TB symptoms at each visit and, in turn, all patients diagnosed with TB are screened for HIV. Clinicians specifically ask for TB symptoms using the World Health Organization (WHO) symptom screening tool [25] and request a chest radiography and sputum smear for bacilloscopy+/- GeneXpert analysis if symptoms of pulmonary tuberculosis are detected. Treatments of both diseases are provided at the same integrated HIV-TB clinic, under the same roof, and by the same medical staff. All staffs at the CDCI underwent training on screening of TB among HIV, HIV among TB, infection control as well as procedural changes at the clinic to accommodate new system changes. In addition, two different waiting areas and entrance to the clinic as well as two different sites for drug dispensing were accommodated to avoid contact between TB and HIV patients.

#### Use of GeneXpert

GeneXpert was introduced in order to improve timely detection and diagnosis of TB among individuals with or without diagnosis of HIV. Sputum or extra-pulmonary samples are obtained from TB suspects when feasible. Results are made available to the clinic within two hours and treatment is started immediately in case of a positive result.

#### Use of electronic data collection system

Previously existing case report forms (CRF) were optimised to include all detailed visit information, concerning medical history, physical examination and diagnoses using International Classification of Diseases (ICD)-10 codes, ART toxicity, drug prescription and refill information, as well as laboratory results. An electronic medical record system was then developed based on these new CRFs using the OpenMRS platform (http://openmrs.org/) and all paper forms were fully replaced. The system, designed to comprehensively capture all clinically relevant information and minimising missing data and data entry mistakes, was launched in December 2012 and has been used without interruption at the CDCI ever since.

### Statistical methods

Baseline characteristics were summarized as proportions/percentages for categorical variables and medians and interquartile ranges for continuous variables. Annual specific notification incidence rates, cumulative incidence rates pre- and during 2013, and incidence rates by selected variables were calculated using Cox proportional hazard model. Estimated number of TB cases, person-years incidence rates and 95% confidence interval (CI) were presented. In addition, the incidence of early tuberculosis ascertainment was assessed by separately calculating the rate of tuberculosis ascertainment in those diagnosed within a month of registration.

Cox proportional hazard models were used to determine independent predictors of tuberculosis ascertainment in order to assess the impact of the bundle of services introduced in December 2012. Hazard ratios, 95% CI, and Wald p values were calculated. Follow-up started at enrolment and patients were censored at the earliest date of the following: first TB diagnosis, last follow-up visit, death, withdrawal from the cohort, or December 31, 2013. We considered events contributing to the follow up if they happen within the period of registration(eg events only count for patients registered prior to 2013 if they happen prior to 2013). We conducted a complete case analysis for all variables in the multivariate analysis and for variables with more than 10% of records missing were assessed in the univariable analysis but not included in the multivariable models. The characteristics of patients with and without missing records for such variables were compared to assess potential systematic differences between patients with and without missing data. The choice of confounders was made based on literature review and clinical knowledgeand all predictors were selected *apriori* and included in the final multivariable model.

Finally, a sensitivity analysis was done in order to estimate the impact of the bundelon early diagnosis and diagnosis on new cases. First we estimated the rate of ascertainment on only patients diagnosed within a month of registration and second estimating rate by excluding those diagnosed within the first month of registration. Data were extracted, processed and analysed using SAS v9.3 (SAS Institute, North Caroline, USA) and STATA v12 (StataCorp, Texas, USA).

## Results

**Fig 1:**
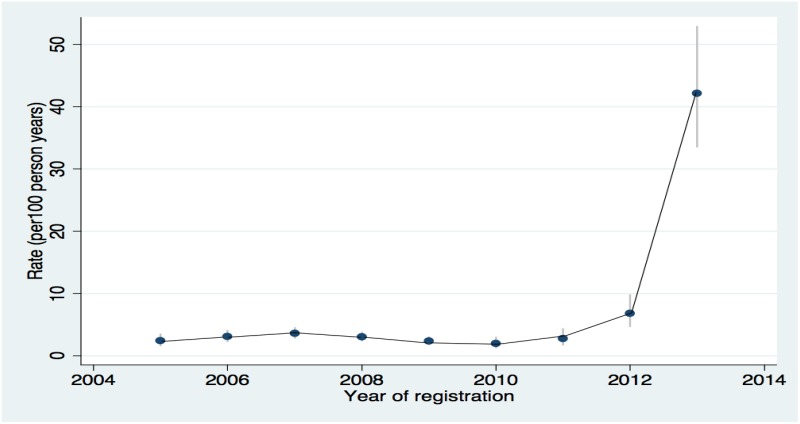
Flow diagram of the study population.

A total of 7114 HIV-infected patients were enrolled in the KIULARCO cohort between January 2005 and December 2013. Of these, 5123 were eligible for the study (Fig 1). At the time of enrolment, the median age was 38 years (Interquartile range (IQR): 30.6, 46) and median CD4 count was 243 cells/μl (IQR: 102, 477). Sixty-six percent were female, 45% were married and 92% were residents of the Kilombero district. Late presentation was common with 27% and 16% enrolling with WHO stage 3 and 4 respectively, and 63% with ≤350 CD4 cells/μl. Six percent of patients were on ART at enrolment and 84% started ART during follow-up, with 80% reporting good adherence. Social demographic and clinical characteristics of the study population did not differ with those of the entire cohort (Table 1).

**Table 1:** Summary of the baseline characteristics of study participants.

	Pre 2013		Post 2013	
Characteristic	Study population (N = 6622)	Eligible population (N = 4772)	Study population (N = 492)	Eligible population (N = 351)
Age (years), median (IQR)	38.1 (30.6–46.1)	38.2 (30.6–46)	36.2 (29.6–45.4)	36 (29.8–45.6)
**Male Gender, n (%)**	2403 (36.6)	1610 (33.9)	168 (34.2)	110 (31.3)
**WHO clinical stage, n (%)**				
Stage III/IV	2204 (52)	1357 (41.8)	265 (55.5)	180 (51.9)
**CD+4 cell count**, **n (%)**				
Median (IQR)cells/ul	228 (91–449)	242 (102.7–477)	201 (66.5–418.5)	201 (67–387)
≤350cells/ul	1501 (65.4)	938 (62.7)	304 (69.1)	230 (71.4)
**On ART during follow-up, n (%)**	4970 (83.7)	4034 (84.6)	301 (61.2)	260 (74.1)
**Functional status**, **n (%)**				
Working	5142 (89.3)	4214 (91.5)	416 (85.1)	313 (89.7)
Ambulatory	502 (8.7)	308 (6.7)	52 (10.6)	25 (7.2)
Bedridden	109 (2)	83 (1.8)	21 (4.3)	11 (3.1)
**Alcohol use, n (%)**	1603 (27.7)	1127 (27.8)	77 (15.6)	53 (15.1)
**Smoking, n (%)**	894 (15.4)	601 (14.8)	14 (2)	8 (2.3)
**History of chronic diseases, n (%)**	942 (15.7)	673 (15.9)	6 (1.2)	4 (1.1)

Percentages exclude missing values: Chronic diseases include arterial hypertension, diabetes mellitus and cancer. KIULARCO: Kilombero Ulanga Antiretroviral Cohort; IQR: Interquartile range; WHO: World Health Organization, ART: Antiretroviral treatment

### Tuberculosis ascertainment

In total 421 TB cases were registered in the KIULARCO cohort between 2005 and 2013. Of these 81 had been registered in 2013, and 47 of the patients registered prior to 2013 developed TB in 2013. Thus, 128/421 (30%) TB cases were diagnosed in 2013. GeneXpert contributed to 12% (15/128) of all TB diagnostics in 2013.

**Fig 2:**
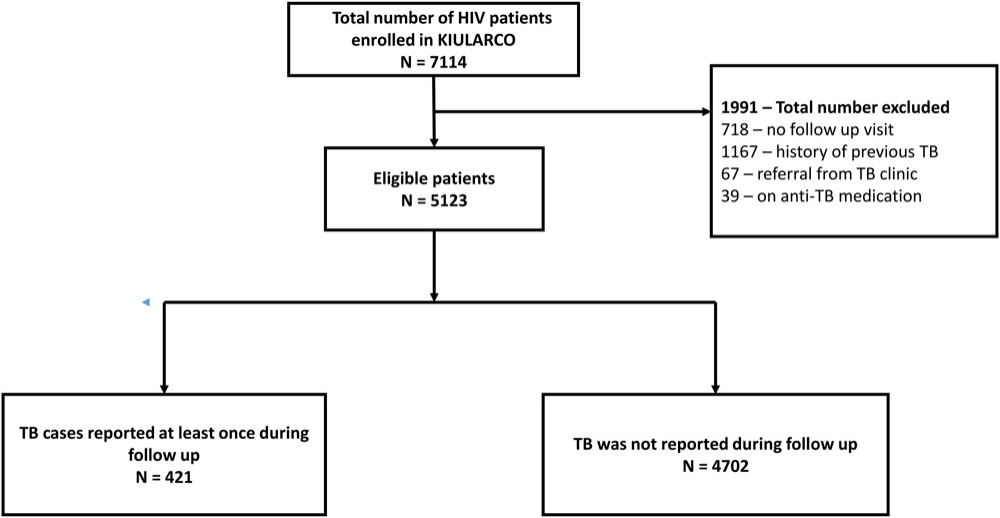
Incidence rate of tuberculosis ascertainment over time, 2005–2013.

The overall person-years (p-y) of follow-up was 11,323 (11,149 p-y prior to 2013 and 175 p-y post 2013) and the incidence rate of TB ascertainment was 3.60 per 100 p-y (95% CI: 3.26–3.97) during the study period. The incidence rate before 2013 was 2.96 per 100 p-y (95% CI: 2.66–3.30) and 43.98 per 100 p-y (95% CI: 35.18–54.99) during 2013 (Fig 2). The incidence rate of TB was higher among males (3.98 per 100 p-y; 95% CI: 3.38–4.67), patients aged<18 years (4.40 per 100 p-y; 95% CI: 3.31–5.83), ART naïve individuals (7.67 per 100 p-y 95%CI 6.28–9.37) and those with baseline WHO clinical stage 3 or 4 (8.30 per 100 p-y; 95% CI: 7.27–9.46, Table 2).

**Table 2:** Incidence rates of Tuberculosis ascertainment among HIV patients enrolled in KIULARCO per selected characteristics.

Characteristics	Overall Incidence rates (per 100 person years)	95% CI	P value	Early incidence rates (per 100 person years)	95% CI	P value	Incidence rate one month after registration (per 100 personyears)	95%CI	P value
**Sex**									
Female	3.43	3.03–3.87	0.06	0.88	0.69–1.12	0.08	2.55	2.21–2.93	0.26
Male	3.98	3.38–4.67		1.23	0.92–1.65		2.75	2.13–3.12	
**Age group**									
Adults ≥18	3.50	3.15–3.89	0.21	0.95	0.78–1.16	0.2	2.55	2.26–2.89	0.48
Children<18	4.40	3.31–5.83		1.37	0.82–2.28		3.02	2.15–4.25	
**WHO clinicalstage**									
1 and 2	2.31	1.91–2.81	<0.0001	0.46	0.30–0.71	<0.0001	1.85	1.49–2.29	<0.0001
3 and 4	8.30	7.27–9.46		2.80	2.23–3.51		5.51	4.68–6.47	
**ART treatment**									
No	7.67	6.28–9.37	<0.0001	4.07	3.10–5.36	<0.0001	3.63	2.71–4.86	0.1
Yes	3.09	2.77–3.46		0.61	0.47–0.78		2.48	2.19–2.81	
**Smoking**									
No	3.36	3.00–3.77	0.92	1.04	0.84–1.28	0.06	2.32	2.02–2.66	0.18
Yes	3.66	2.73–4.91		0.57	0.27–1.19		3.10	2.26–4.27	
**History of any chronic disease**									
No	3.32	2.97–3.72	0.32	0.97	0.78–1.19	0.39	2.36	2.06–2.70	0.54
Yes	3.63	2.63–5.01		1.07	0.59–1.94		2.56	1.74–3.76	
**Periodofregistration (years)**									
Before 2013	2.96	2.66–3.30	<0.0001	0.57	0.44–0.73	<0.0001	2.39	1.93–2.46	<0.0001
2013	43.98	35.18–54.99		27.99	21.15–37.03		16.36	11.29–23.69	

Chronic diseases include arterial hypertension, diabetes mellitus and cancer. TB: tuberculosis; Confidence interval; WHO: World Health Organization; ART: Antiretroviral treatment

During the same period,1868 TB cases were reported from Saint Francis Referral Hospital to the National Tuberculosis and leprosy programme (NTLP). Of them, 1363 (73%) were pulmonary TB and 505 (27%) extrapulmonary. Only 27% of pulmonary cases were confirmed through microscopy smear. In 2013, 84% (141/167) of all TB/HIV cases reported to the NTLP from our hospital were captured in the KIULARCO database as compared with 16% (280/1701) prior to 2013.

### Predictors of tuberculosis ascertainment

In univariate analysis, being single, WHO clinical stage 3 or 4, having a poor functional status at baseline (being bedridden or unable to work), CD4 count ≤350 cells/μl, ART-naivety, and registration in 2013 versus pre-2013 were associated with an increased rate of TB recording in the KIULARCO database.

Four baseline independent predictors of increased risk of TB ascertainment were identified in the multivariate analysis: registration in 2013 (HR 6.07, 95% CI: 4.39–8.38), WHO stage 3 or 4 (HR 2.48, 95% CI: 1.88–3.26), poor functional status at baseline (HR 3.89, 95% CI; 2.87–5.28), and ART-naivety (HR 2.97, 95%CI 2.25–3.94), Table 3).

**Table 3:** Cox regression analysis of risk factors for tuberculosis ascertainment by period.

	2005–2013		Pre-2013		Post 2013	
Characteristics	Unadjusted hazard ratio (95% CI)	Adjusted hazard ratio (95%CI)	Unadjusted hazard ratio (95% CI)	Adjusted hazard ratio (95%CI)	Unadjusted hazard ratio (95% CI)	Adjusted hazard ratio (95%CI)
**Gender**						
Female	1	1	1	1	1	1
Male	1.20 (0.98–1.48)	1.12 (1.85–1.47)	1.11 (0.89–1.40)	0.93 (0.67–1.31)	1.97 (1.25–3.09)	1.72 (1.02–2.87)
**Age group (years)**						
≥18	1	1	1	1	1	1
<18	1.31 (1.07–1.61)	1.02 (0.68–1.50)	1.03 (0.72–1.46)	1.03 (0.65–1.65)	2.67 (1.50–4.80)	1.24 (0.57–2.68)
**Marital status**						
Married	1	1	1	1		1
Not Married	1.31 (1.07–1.61)	1.27(0.97–1.66)	1.21 (0.96–1.52)	1.15 (0.84–1.57)	2.41 (1.48–3.90)	1.35 (0.77–2.34)
**WHO stage**						
I/II	1	1	1	1	1	1
III/IV	3.22 (2.54–4.06)	2.48 (1.88–3.26)	2.82 (2.17–3.66)	1.94 (1.43–2.65)	4.50 (2.58–7.83)	3.97 (2.14–7.32)
**ART**						
Yes	1	1	1	1	1	1
No	2.12 (1.69–2.67)	2.97 (2.25–3.94)	1.12 (0.82–1.53)	1.42 (0.94–2.13)	9.35 (5.80–15.1)	11.1 (6.5–1874)
**Smoking**						
No	1	1	1	1	1	1
Yes	1.01 (0.74–1.39)	1.21 (0.81–1.82)	1.27 (0.91–1.77)	1.28 (0.83–1.96)	1.20 (0.29–4.90)	1.90 (0.43–8.34)
**Chronic disease¤**						
No	1	1	1	1	1	1
Yes	6.30 (4.83–8.21)	1.36 (0.92–1.99)	1.09 (0.76–1.56)	1.50 (1.01–2.21)	0.91 (0.13–6.52)	1.50 (0.19–11.27)
**Functional status**						
Working	1	1	1	1	1	
Ambulatory or bedridden	6.20 (4.90–7.83)	3.89 (2.87–5.28)	6.59 (5.07–8.57)	4.80 (3.36–6.83)	4.44 (2.64–7.46)	
**Registration (year)**						
Before 2013	1	1				
2013	6.30 (4.83–8.21)	6.07 (4.39–8.38)				

### Early tuberculosis diagnosis

In total 126 TB cases were recorded in KIULARCO within a month after enrolment, 73 pre- and 53 post-2012. Seventy-five percent (55/73) and 81% (43/53) of those diagnosed before and after 2012 respectively were ART-naïve. The overall rate of early diagnosis pre-2013 was 0.57 cases per 100 p-y (95% CI 0.44–0.73) and 28 per 100 p-y (95% CI 21.15–37.03) in 2013 (p<0.0001). Early diagnosis was higher among ART-naive compared to those on ART (4.07 per 100 p-y (95% CI 3.10–5.36) vs 0.61 per 100 p-y (95% CI 0.47–0.78) respectively). Independent predictors of early diagnosis did not differ from those of the entire cohort.

## Discussion

In this large study including 5123 HIV infected patients enrolled in a rural African HIV prospective cohort between 2005 and 2013, the previously stable TB incidence rate dramatically increased after the introduction of a bundle of services consisting of the integration of HIV and TB services within one single facility, use of GeneXpert, and implementation of an electronic data collection system. In addition, the bundle of services had an impact on early detection of TB among ART naïve patients. Moreover the strongest predictor of TB identified was registration in the clinic after the introduction of the bundle of services. To our knowledge this is the first report to show the impact of such a bundle of services on tuberculosis ascertainment in Africa. Previous studies on trends of TB case notification in Africa have indicated a decrease in trend among HIV patients following scaling up of ART [5,6]. We implemented system changes which emphasize intensification of screening for TB among HIV patients and optimized data recording which resulted into identifying more TB incident cases. Thus, our findings suggest a potential under-diagnosis and/or under-reporting of TB among HIV infected individuals in similar rural African settings.

Our findings concur with previous studies from sub-Saharan Africa showing an increase of TB diagnosis when TB and HIV programs are integrated and TB screening intensified[9–11]. Integration of both programs in one single health facility provides an opportunity for early diagnosis of HIV among TB patients as well as early diagnosis of TB among HIV patients[9]. Different models have been implemented to integrate TB and HIV care, ranging from models including referrals between the two clinics to more comprehensive models offering care under one facility[12]. The latter, though demanding in organizational and administrative structure, has proved to be more effective [12]. However, some challenges exist including training and retention of staff, and infection control[12].

GeneXpert has been shown to have a high sensitivity and specificity for TB diagnosis [7,13]. Previous studies in high burden countries have indicated an increase in TB case reporting when GeneXpert was used compared to sputum microscopy[7,14–16]. Vassall A (2011) et al observed an overall increase in TB detection in India, South Africa and Uganda from 72%-85% to 95%-99% [14]. Results from a decision model study from South Africa on impact of national scaling up suggested a potential 30%-37% increase in the number of TB cases diagnosed per year[17]. Another study in the same setting indicated an increase of 45% in HIV associated TB detection when GeneXpert was used compared to smear microscopy[15]. However, the value of GeneXpert in high TB burden settings where empirical treatment is common has been recently questioned, suggesting that in these settings wide GeneXpert use may displace empirical treatment instead of improving outcomes and disease burden [18]. Empirical treatment at the CDCI was initiated after negative microbiological tests, a course of antibiotics without effect, and a radiography suggestive of TB. We observed an increase of almost fifteen-fold in the rate of TB ascertainment after introduction of the bundle of services including GeneXpert use. However, our findings of an increased TB ascertainment do not seem to be fully explained by the use of GeneXpert, since it only accounted for 12% of additional TB cases detected in 2013.

Previous studies have reported that the use of electronic system in medical records help improve health care quality in general and for HIV/AIDS patients [19–22]. We replaced the previous paper-based system with the electronic system, which prompts clinicians to inquire and report all required information. Specifically, clinicians are compelled to collect information on the screening of symptoms and signs of TB in every visit and report the cases using ICD-10 codes. TB screening among all HIV-infected individuals attending the clinic is currently performed at all visits and all TB patients are in turn routinely screened for HIV. This suggests that the observed increase in incidence rates of TB ascertainment was partly due to changes in recording systems as well as improved clinical information collection tools. Indeed, 84% of all TB/HIV cases recorded at the National Tuberculosis and Leprosy Programme (NTLP) from our hospital were captured in the KIULARCO database 2013a 68% increase from the period prior to 2013.

There are limited data on the incidence of TB notification among HIV patients in Tanzania. The latest estimates of prevalence of TB by the WHO and the Tanzanian Ministry of Health are 177 per 100,000 persons and 295 per 100,000 persons respectively among the general population [26,27]. In 2013, we obtained an overall estimated incidence notification rate of 44 cases per 100 p-y. Due to the lack of comparative studies in similar populations, our findings provide relevant information to assess the incidence rates of TB among HIV patients in Tanzania. However, this incidence rate is high compared to previous estimations from South Africa (9.2 cases per 100 person-years)[28]. Importantly, despite acknowledging a potential overestimation, our findings reflect the incidence of tuberculosis in a “real-life” rural African setting, with a high proportion of clinical diagnosis and empiric treatment. The incidence of TB diagnosis in children was estimated to be 4.4 per 100 p-y slightly, similar to previous results from Dar es Salaam (5.2 per 100 p-y)[29]. As expected, we observed a lower rate of TB notification among those who were on ART compared to ART-naive patients, concurring with a number of studies showing reduction in the risk of TB among HIV patients on ART[30–35]. Remarkably, we also observed an increase in early TB notification after implementing system changes. Eighty one percent of patients diagnosed early in 2013 were ART naïve at the time of TB diagnosis, compared to 75% pre-2013. Increasing TB notification among ART-naïve individuals has important clinical implications, reducing the incidence of immune reconstitution inflammatory syndrome (IRIS), and increasing survival[36].

Four baseline factors were identified as independent predictors of TB, including WHO clinical stage 3 or 4, ART naivety, poor functional status, and registration in 2013. There were not remarkable differences in predictors of TB ascertainment between pre- and post 2013. Our findings are similar to those by Illiyasu Z *et al*. in which marital status, WHO stage and CD4+ count predicted TB occurrence [37]. We did not include CD4+ count in the multivariable model due to significant missing values, which would have resulted into an unstable model. However, we presented the univariable estimates that suggested that CD4+ count was an important predictor of TB notification in our population. An attempted sensitivity analysis by including missing values on CD4+ count through imputation of the mean did not modify the results and the rate of TB notification was still higher among those with CD4+ count ≤350cells/μl. Remarkably, registration in 2013 was the strongest predictor of TB notification even after excluding early TB notification in sensitivity analysis, suggesting a critical role of the bundle of services introduced in December 2012.

This study has certain limitations. First, due to the combined introduction of the three interventions, the improvement in TB ascertainment could not be attributed to any particular intervention but to the bundle of services. However, supporting other observations, GeneXpert does not seem to have played a prominent role in increasing TB ascertainment. Second, a longer follow-up will be needed to measure the long-term impact of this strategy. Despite the extent of missing values on baseline WHO clinical stage and CD4+ count being more than 10%, it is unlikely that our study population was biased due to missing data since the characteristics of the study population and that of the whole cohort did not differ. Third, we acknowledge that the influence of other factors such as changes in clinical administration and motivation of clinicians may have contributed. However, these factors are not easily measured. Fourth, our findings indicate an increase in diagnosis of TB within the KIULARCO cohort, but it has been difficult to estimate accurately whether the impact of this bundle of services also resulted in an increased notification to the National Tuberculosis and Leprosy Program. Finally, we recognize the potential for information bias and record error, since tuberculosis cases for this study were entirely based on records in the database.

There are several clinical and public health implications of our findings, particularly in light of the striking disparity in TB ascertainment and reporting before and after the implementation of the bundle of services in our clinic. First, our results support the need for a switch from vertical models to comprehensive care models in rural sub-Saharan Africa. The integration of TB and HIV services within one single facility is a first step in this direction. This integration allowed for the systematic application of diagnostic algorithms including clinical information, chest radiography, and a true point-of-care use of GeneXpert, providing results in less than two hours, and decreasing the waiting time and travel expenses for our patients. GeneXpert is being progressively rolled out in sub-Saharan Africa, but as important as its roll out is the adaptation of the health facilities to this new technology and its full integration within the existing routine diagnostic algorithms to maximize its potential benefits. Second, our findings stress the several advantages of the replacement of clinic logs with electronic medical records, which we believe should be a public health priority in these settings to optimize clinical efficiency and epidemiological surveillance. Finally our results suggest the possibility of an under-ascertainment of TB-HIV in similar settings in Tanzania and other sub-Saharan African countries. TB is the first cause of death among HIV-infected patients in Africa, and can be fatal if non-diagnosed and treated timely. Moreover, ART initiation in non-diagnosed active TB cases may result in the development of unmasking TB-IRIS, posing a tremendous clinical challenge to clinicians in these settings and carrying significant morbidity and mortality if not properly managed.

In summary, we believe that integrating tuberculosis and HIV services together with a comprehensive electronic data collection and use of appropriate diagnostic tools are pivotal in assessing the burden of tuberculosis among HIV-positive patients and may contribute to decrease the unacceptably high HIV-TB-associated mortality in Africa. Further studies to assess the potential effects of similar strategies on this and other clinical outcomes are warranted.
