# Correction: A Bundle of Services Increased Ascertainment of Tuberculosis among HIV-Infected Individuals Enrolled in a HIV Cohort in Rural Sub-Saharan Africa

**DOI:** 10.1371/journal.pone.0129185

**Published:** 2015-05-20

**Authors:** Frederick Haraka, Tracy R. Glass, George Sikalengo, Anna Gamell, Alex Ntamatungiro, Christoph Hatz, Marcel Tanner, Hansjakob Furrer, Manuel Battegay, Emilio Letang

The images for Figs 1 and 2 are incorrectly switched. The image that appears as Fig 1 should be Fig 2, and the image that appears as Fig 2 should be Fig 1. The figure captions appear in the correct order.

**Fig 1:**
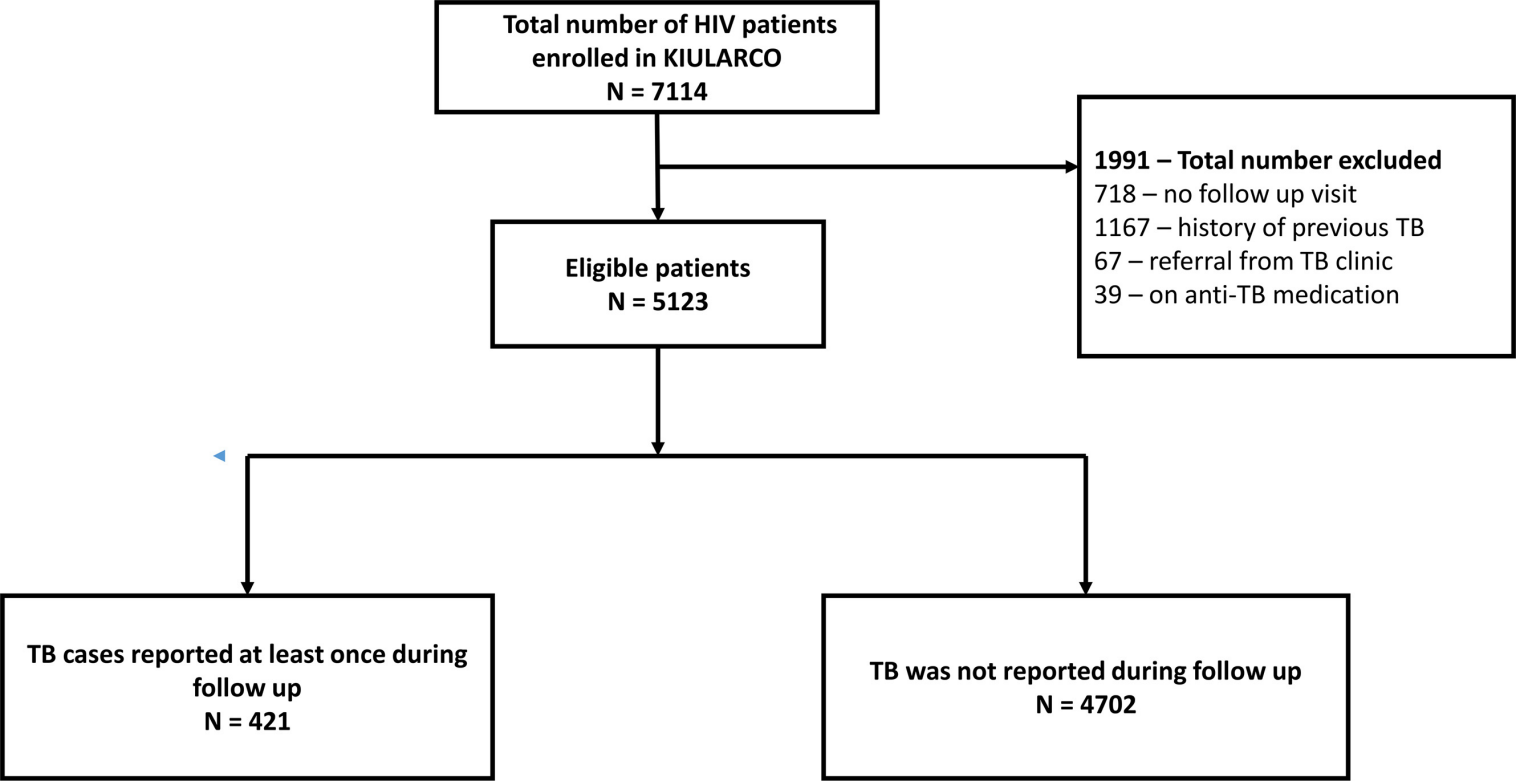
Flow diagram of the study population.

**Fig 2:**
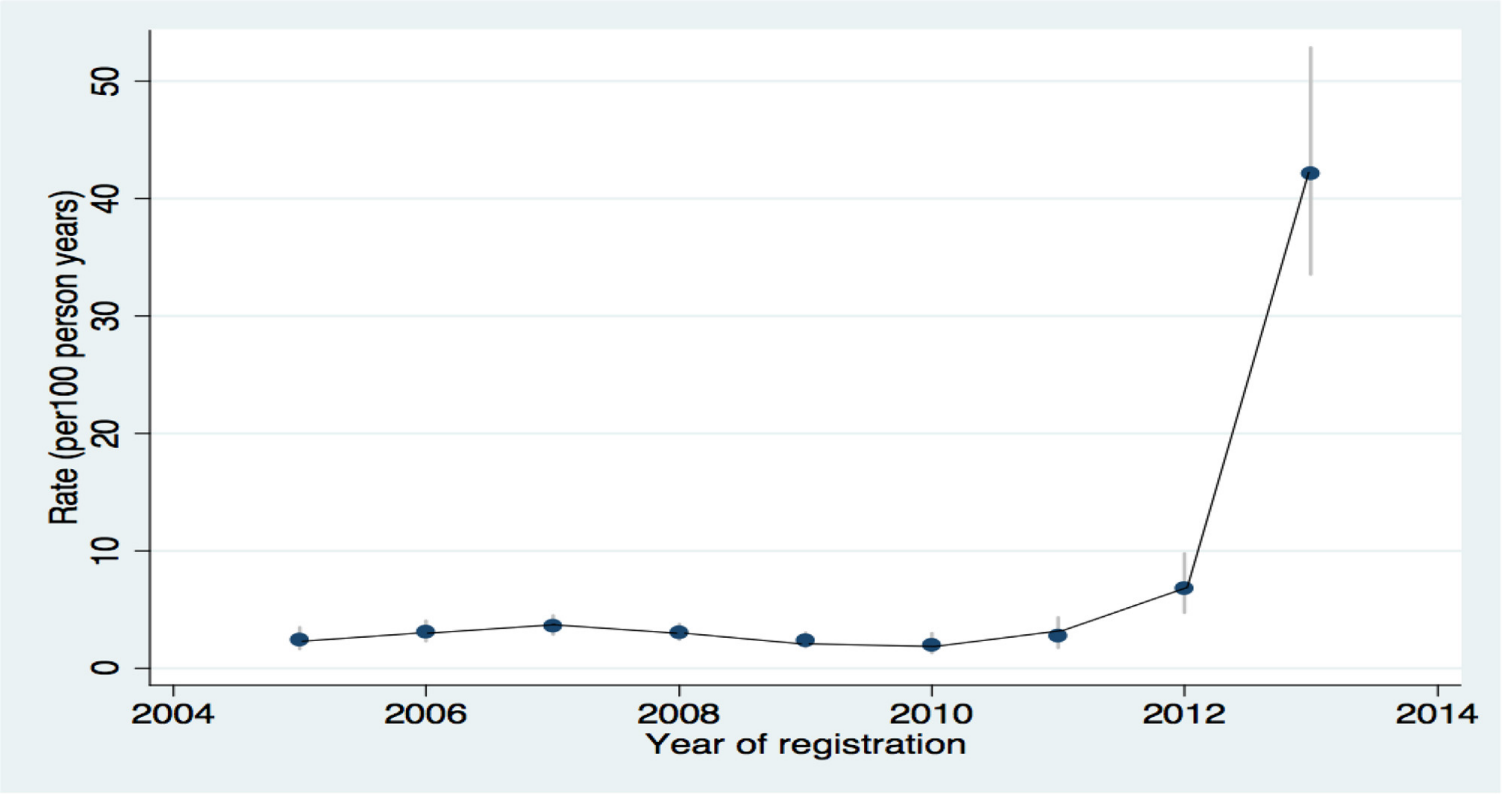
Incidence rate of tuberculosis ascertainment over time, 2005–2013.

## References

[1] Haraka F, Glass TR, Sikalengo G, Gamell A, Ntamatungiro A, Hatz C, et al (2015) A Bundle of Services Increased Ascertainment of Tuberculosis among HIV-Infected Individuals Enrolled in a HIV Cohort in Rural Sub-Saharan Africa. PLoS ONE 10(4): e0123275 doi: 10.1371/journal.pone.0123275 25897491 10.1371/journal.pone.0123275 PMC4405488

